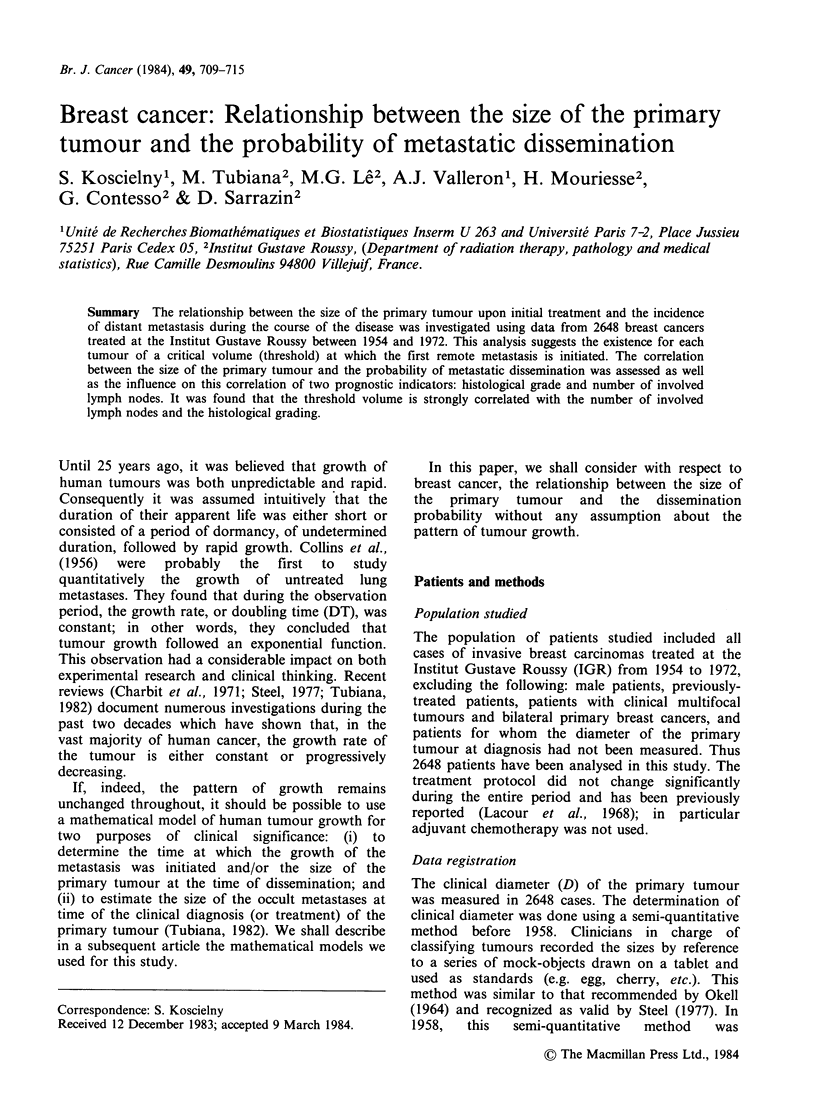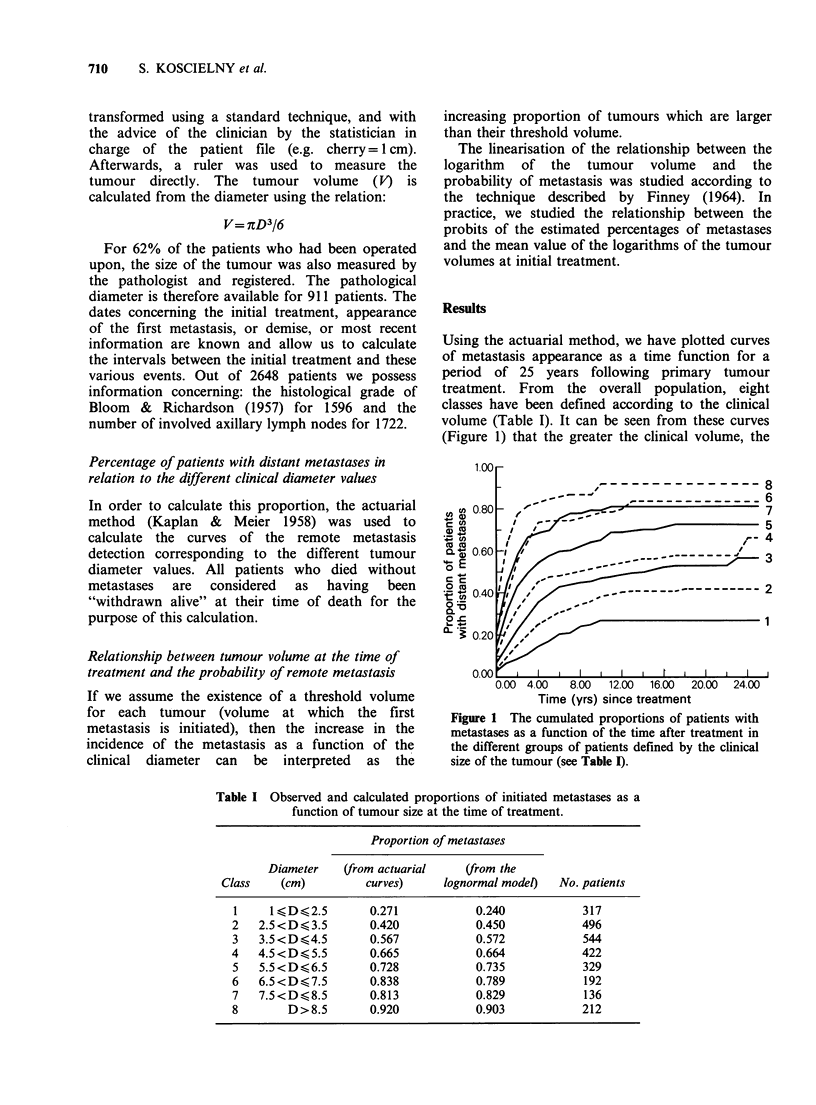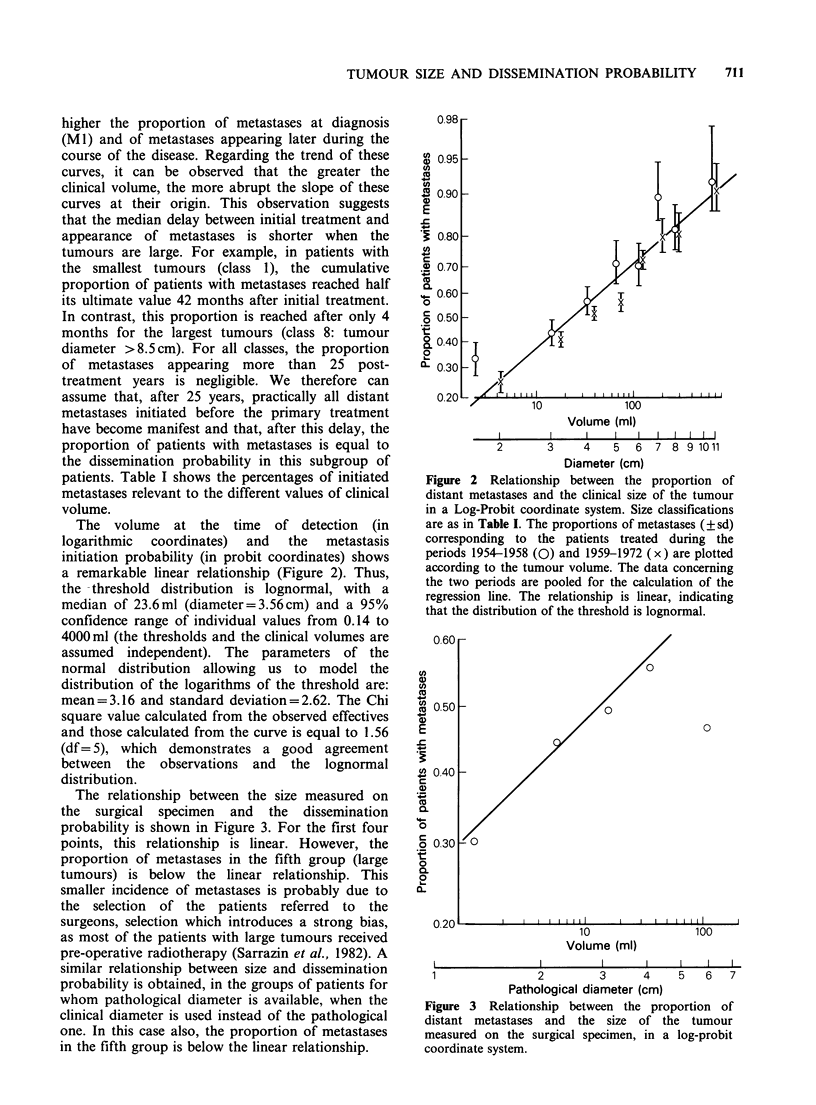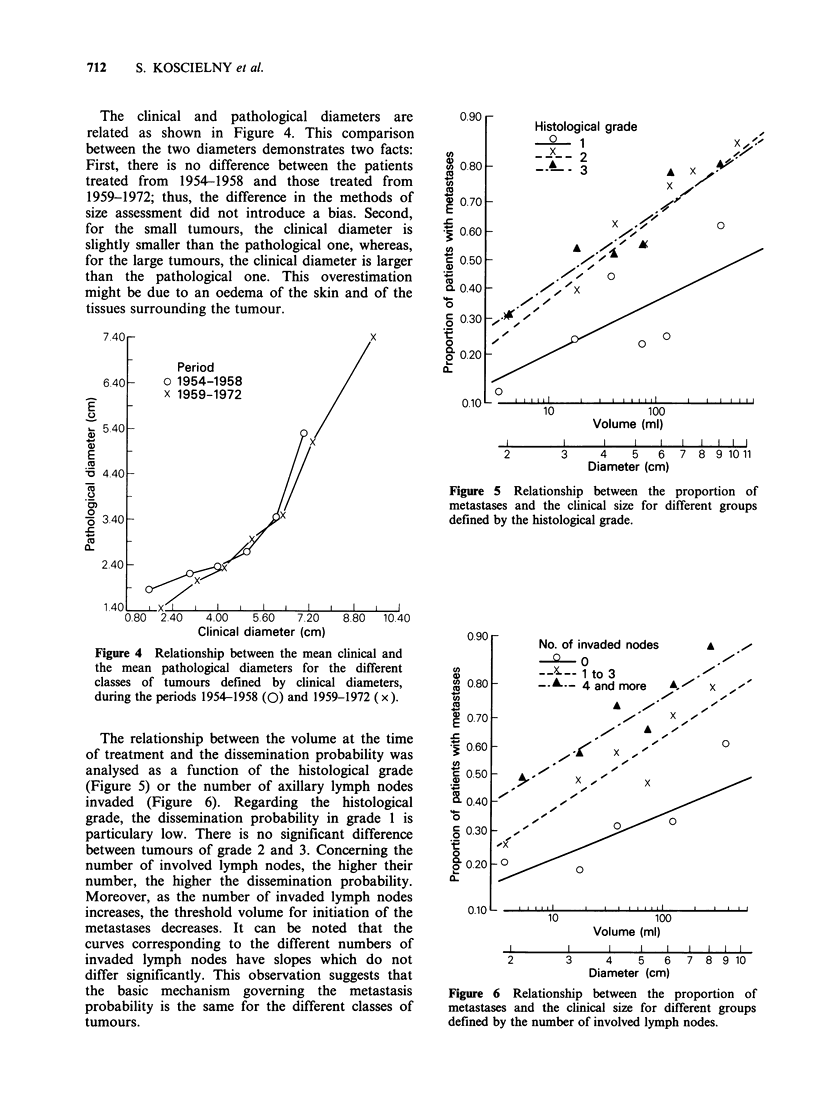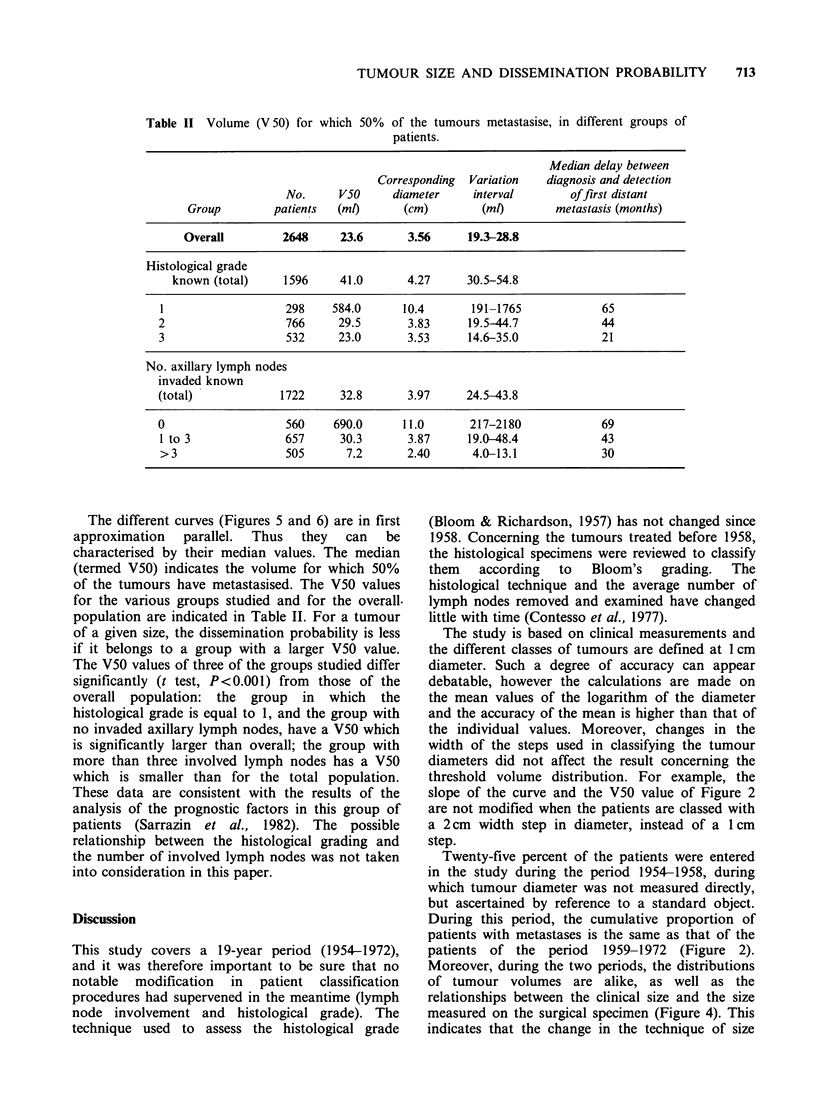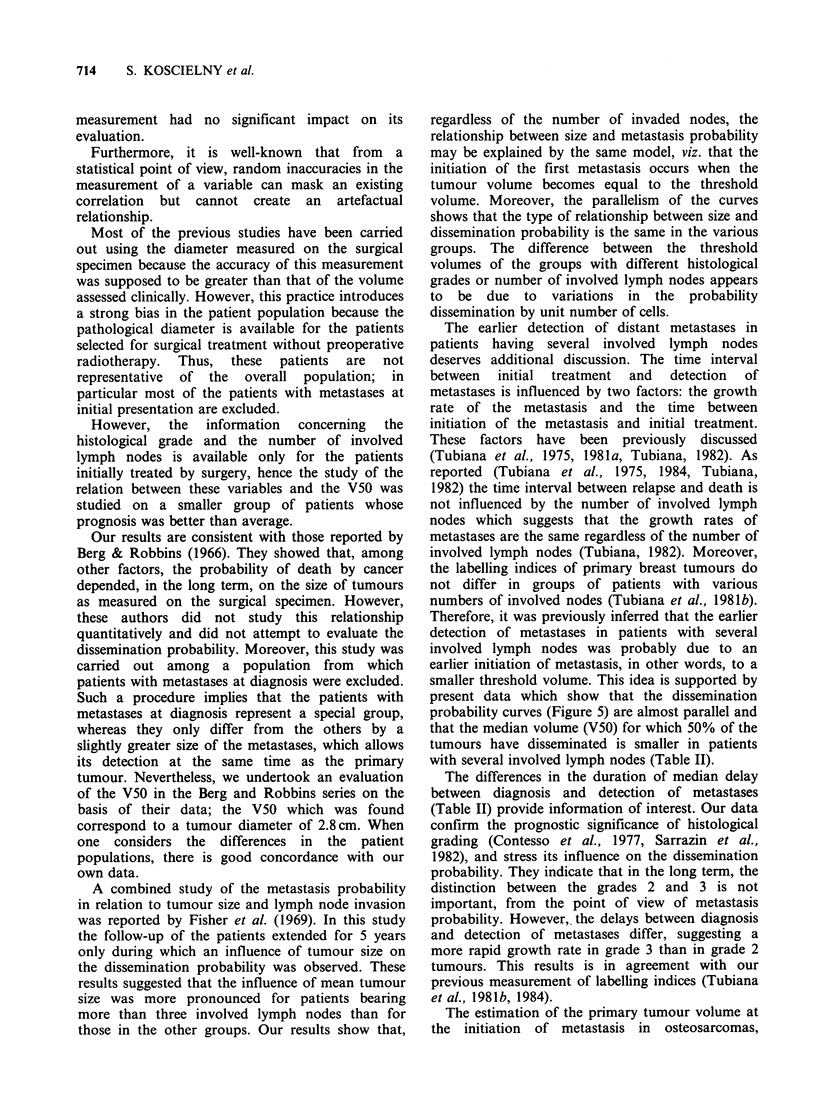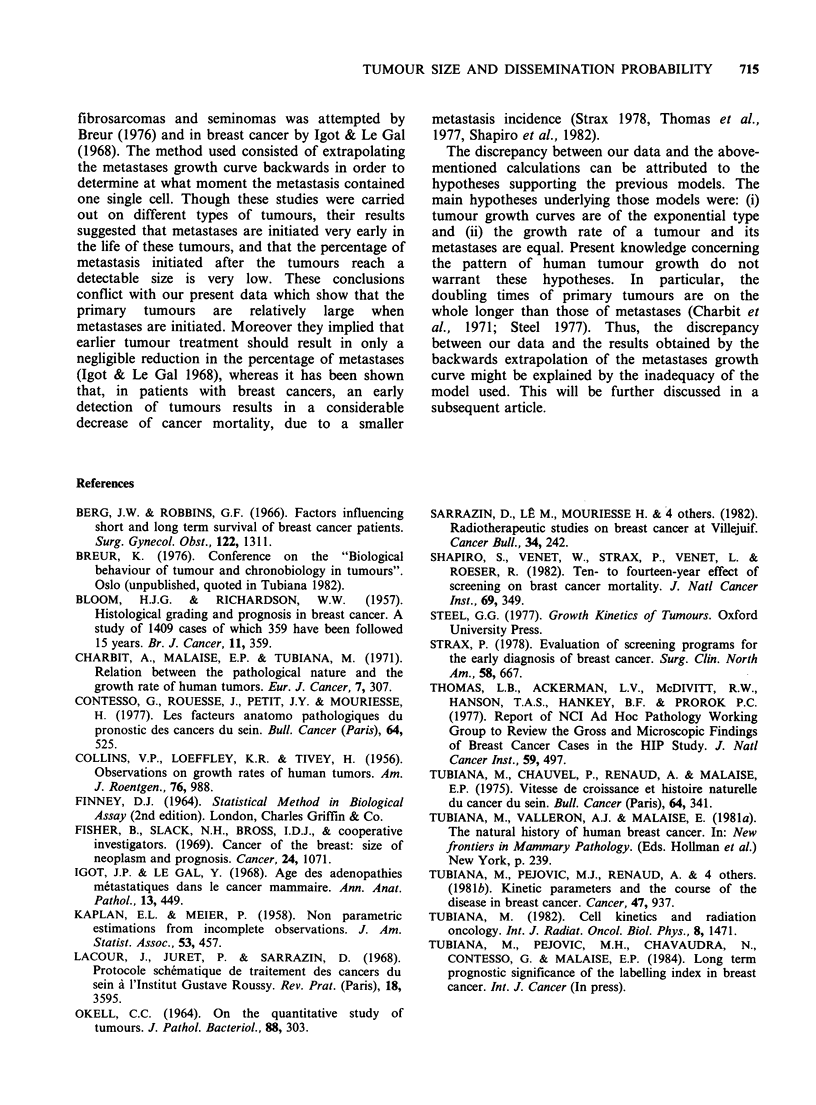# Breast cancer: relationship between the size of the primary tumour and the probability of metastatic dissemination.

**DOI:** 10.1038/bjc.1984.112

**Published:** 1984-06

**Authors:** S. Koscielny, M. Tubiana, M. G. Lê, A. J. Valleron, H. Mouriesse, G. Contesso, D. Sarrazin

## Abstract

The relationship between the size of the primary tumour upon initial treatment and the incidence of distant metastasis during the course of the disease was investigated using data from 2648 breast cancers treated at the Institut Gustave Roussy between 1954 and 1972. This analysis suggests the existence for each tumour of a critical volume (threshold) at which the first remote metastasis is initiated. The correlation between the size of the primary tumour and the probability of metastatic dissemination was assessed as well as the influence on this correlation of two prognostic indicators: histological grade and number of involved lymph nodes. It was found that the threshold volume is strongly correlated with the number of involved lymph nodes and the histological grading.


*Br. J. Cancer* (1984), **49**, 709–715

### Breast cancer: Relationship between the size of the primary tumour and the probability of metastatic dissemination

S. Koscielny[1], M. Tubiana[2], M.G. Lê[2], A.J. Valleron[1], H. Mouriesse[2], G. Contesso[2] & D. Sarrazin[2]

[1]*Unité de Recherches Biomathématiques et Biostatistiques Inserm U 263 and Université Paris 7-2, Place Jussieu 75251 Paris Cedex 05,* [2]*Institut Gustave Roussy, (Department of radiation therapy, pathology and medical statistics), Rue Camille Desmoulins 94800 Villejuif, France.*

**Summary** The relationship between the size of the primary tumour upon initial treatment and the incidence of distant metastasis during the course of the disease was investigated using data from 2648 breast cancers treated at the Institut Gustave Roussy between 1954 and 1972. This analysis suggests the existence for each tumour of a critical volume (threshold) at which the first remote metastasis is initiated. The correlation between the size of the primary tumour and the probability of metastatic dissemination was assessed as well as the influence on this correlation of two prognostic indicators: histological grade and number of involved lymph nodes. It was found that the threshold volume is strongly correlated with the number of involved lymph nodes and the histological grading.

Until 25 years ago, it was believed that growth of human tumours was both unpredictable and rapid. Consequently it was assumed intuitively that the duration of their apparent life was either short or consisted of a period of dormancy, of undetermined duration, followed by rapid growth. Collins *et al.*, (1956) were probably the first to study quantitatively the growth of untreated lung metastases. They found that during the observation period, the growth rate, or doubling time (DT), was constant; in other words, they concluded that tumour growth followed an exponential function. This observation had a considerable impact on both experimental research and clinical thinking. Recent reviews (Charbit *et al.*, 1971; Steel, 1977; Tubiana, 1982) document numerous investigations during the past two decades which have shown that, in the vast majority of human cancer, the growth rate of the tumour is either constant or progressively decreasing.

If, indeed, the pattern of growth remains unchanged throughout, it should be possible to use a mathematical model of human tumour growth for two purposes of clinical significance: (i) to determine the time at which the growth of the metastasis was initiated and/or the size of the primary tumour at the time of dissemination; and (ii) to estimate the size of the occult metastases at time of the clinical diagnosis (or treatment) of the primary tumour (Tubiana, 1982). We shall describe in a subsequent article the mathematical models we used for this study.

Correspondence: S. Koscielny
Received 12 December 1983; accepted 9 March 1984.

In this paper, we shall consider with respect to breast cancer, the relationship between the size of the primary tumour and the dissemination probability without any assumption about the pattern of tumour growth.

### Patients and methods

### *Population studied*

The population of patients studied included all cases of invasive breast carcinomas treated at the Institut Gustave Roussy (IGR) from 1954 to 1972, excluding the following: male patients, previously-treated patients, patients with clinical multifocal tumours and bilateral primary breast cancers, and patients for whom the diameter of the primary tumour at diagnosis had not been measured. Thus 2648 patients have been analysed in this study. The treatment protocol did not change significantly during the entire period and has been previously reported (Lacour *et al.*, 1968); in particular adjuvant chemotherapy was not used.

### *Data registration*

The clinical diameter (*D*) of the primary tumour was measured in 2648 cases. The determination of clinical diameter was done using a semi-quantitative method before 1958. Clinicians in charge of classifying tumours recorded the sizes by reference to a series of mock-objects drawn on a tablet and used as standards (e.g. egg, cherry, *etc.*). This method was similar to that recommended by Okell (1964) and recognized as valid by Steel (1977). In 1958, this semi-quantitative method was

© The Macmillan Press Ltd., 1984

transformed using a standard technique, and with the advice of the clinician by the statistician in charge of the patient file (e.g. cherry = 1 cm). Afterwards, a ruler was used to measure the tumour directly. The tumour volume ($V$) is calculated from the diameter using the relation:

$$V = \pi D^3/6$$

For 62% of the patients who had been operated upon, the size of the tumour was also measured by the pathologist and registered. The pathological diameter is therefore available for 911 patients. The dates concerning the initial treatment, appearance of the first metastasis, or demise, or most recent information are known and allow us to calculate the intervals between the initial treatment and these various events. Out of 2648 patients we possess information concerning: the histological grade of Bloom & Richardson (1957) for 1596 and the number of involved axillary lymph nodes for 1722.

*Percentage of patients with distant metastases in relation to the different clinical diameter values*

In order to calculate this proportion, the actuarial method (Kaplan & Meier 1958) was used to calculate the curves of the remote metastasis detection corresponding to the different tumour diameter values. All patients who died without metastases are considered as having been "withdrawn alive" at their time of death for the purpose of this calculation.

*Relationship between tumour volume at the time of treatment and the probability of remote metastasis*

If we assume the existence of a threshold volume for each tumour (volume at which the first metastasis is initiated), then the increase in the incidence of the metastasis as a function of the clinical diameter can be interpreted as the increasing proportion of tumours which are larger than their threshold volume.

The linearisation of the relationship between the logarithm of the tumour volume and the probability of metastasis was studied according to the technique described by Finney (1964). In practice, we studied the relationship between the probits of the estimated percentages of metastases and the mean value of the logarithms of the tumour volumes at initial treatment.

### Results

Using the actuarial method, we have plotted curves of metastasis appearance as a time function for a period of 25 years following primary tumour treatment. From the overall population, eight classes have been defined according to the clinical volume (Table I). It can be seen from these curves (Figure 1) that the greater the clinical volume, the

**Figure 1** The cumulated proportions of patients with metastases as a function of the time after treatment in the different groups of patients defined by the clinical size of the tumour (see **Table I**).

**Table I** Observed and calculated proportions of initiated metastases as a function of tumour size at the time of treatment.

| Class | Diameter (cm) | Proportion of metastases (from actuarial curves) | (from the lognormal model) | No. patients |
|---|---|---|---|---|
| 1 | 1⩽D⩽2.5 | 0.271 | 0.240 | 317 |
| 2 | 2.5<D⩽3.5 | 0.420 | 0.450 | 496 |
| 3 | 3.5<D⩽4.5 | 0.567 | 0.572 | 544 |
| 4 | 4.5<D⩽5.5 | 0.665 | 0.664 | 422 |
| 5 | 5.5<D⩽6.5 | 0.728 | 0.735 | 329 |
| 6 | 6.5<D⩽7.5 | 0.838 | 0.789 | 192 |
| 7 | 7.5<D⩽8.5 | 0.813 | 0.829 | 136 |
| 8 | D>8.5 | 0.920 | 0.903 | 212 |

higher the proportion of metastases at diagnosis (M1) and of metastases appearing later during the course of the disease. Regarding the trend of these curves, it can be observed that the greater the clinical volume, the more abrupt the slope of these curves at their origin. This observation suggests that the median delay between initial treatment and appearance of metastases is shorter when the tumours are large. For example, in patients with the smallest tumours (class 1), the cumulative proportion of patients with metastases reached half its ultimate value 42 months after initial treatment. In contrast, this proportion is reached after only 4 months for the largest tumours (class 8: tumour diameter >8.5 cm). For all classes, the proportion of metastases appearing more than 25 post-treatment years is negligible. We therefore can assume that, after 25 years, practically all distant metastases initiated before the primary treatment have become manifest and that, after this delay, the proportion of patients with metastases is equal to the dissemination probability in this subgroup of patients. Table I shows the percentages of initiated metastases relevant to the different values of clinical volume.

The volume at the time of detection (in logarithmic coordinates) and the metastasis initiation probability (in probit coordinates) shows a remarkable linear relationship (Figure 2). Thus, the threshold distribution is lognormal, with a median of 23.6 ml (diameter = 3.56 cm) and a 95% confidence range of individual values from 0.14 to 4000 ml (the thresholds and the clinical volumes are assumed independent). The parameters of the normal distribution allowing us to model the distribution of the logarithms of the threshold are: mean = 3.16 and standard deviation = 2.62. The Chi square value calculated from the observed effectives and those calculated from the curve is equal to 1.56 (df = 5), which demonstrates a good agreement between the observations and the lognormal distribution.

The relationship between the size measured on the surgical specimen and the dissemination probability is shown in Figure 3. For the first four points, this relationship is linear. However, the proportion of metastases in the fifth group (large tumours) is below the linear relationship. This smaller incidence of metastases is probably due to the selection of the patients referred to the surgeons, selection which introduces a strong bias, as most of the patients with large tumours received pre-operative radiotherapy (Sarrazin *et al.*, 1982). A similar relationship between size and dissemination probability is obtained, in the groups of patients for whom pathological diameter is available, when the clinical diameter is used instead of the pathological one. In this case also, the proportion of metastases in the fifth group is below the linear relationship.

**Figure 2** Relationship between the proportion of distant metastases and the clinical size of the tumour in a Log-Probit coordinate system. Size classifications are as in **Table I**. The proportions of metastases (±sd) corresponding to the patients treated during the periods 1954–1958 (○) and 1959–1972 (×) are plotted according to the tumour volume. The data concerning the two periods are pooled for the calculation of the regression line. The relationship is linear, indicating that the distribution of the threshold is lognormal.

**Figure 3** Relationship between the proportion of distant metastases and the size of the tumour measured on the surgical specimen, in a log-probit coordinate system.

The clinical and pathological diameters are related as shown in Figure 4. This comparison between the two diameters demonstrates two facts: First, there is no difference between the patients treated from 1954–1958 and those treated from 1959–1972; thus, the difference in the methods of size assessment did not introduce a bias. Second, for the small tumours, the clinical diameter is slightly smaller than the pathological one, whereas, for the large tumours, the clinical diameter is larger than the pathological one. This overestimation might be due to an oedema of the skin and of the tissues surrounding the tumour.

**Figure 4** Relationship between the mean clinical and the mean pathological diameters for the different classes of tumours defined by clinical diameters, during the periods 1954–1958 (O) and 1959–1972 (×).

The relationship between the volume at the time of treatment and the dissemination probability was analysed as a function of the histological grade (Figure 5) or the number of axillary lymph nodes invaded (Figure 6). Regarding the histological grade, the dissemination probability in grade 1 is particulary low. There is no significant difference between tumours of grade 2 and 3. Concerning the number of involved lymph nodes, the higher their number, the higher the dissemination probability. Moreover, as the number of invaded lymph nodes increases, the threshold volume for initiation of the metastases decreases. It can be noted that the curves corresponding to the different numbers of invaded lymph nodes have slopes which do not differ significantly. This observation suggests that the basic mechanism governing the metastasis probability is the same for the different classes of tumours.

**Figure 5** Relationship between the proportion of metastases and the clinical size for different groups defined by the histological grade.

**Figure 6** Relationship between the proportion of metastases and the clinical size for different groups defined by the number of involved lymph nodes.

**Table II** Volume (V 50) for which 50% of the tumours metastasise, in different groups of patients.

| *Group* | *No. patients* | *V50 (ml)* | *Corresponding diameter (cm)* | *Variation interval (ml)* | *Median delay between diagnosis and detection of first distant metastasis (months)* |
|---|---|---|---|---|---|
| **Overall** | **2648** | **23.6** | **3.56** | **19.3–28.8** | |
| Histological grade known (total) | 1596 | 41.0 | 4.27 | 30.5–54.8 | |
| 1 | 298 | 584.0 | 10.4 | 191–1765 | 65 |
| 2 | 766 | 29.5 | 3.83 | 19.5–44.7 | 44 |
| 3 | 532 | 23.0 | 3.53 | 14.6–35.0 | 21 |
| No. axillary lymph nodes invaded known (total) | 1722 | 32.8 | 3.97 | 24.5–43.8 | |
| 0 | 560 | 690.0 | 11.0 | 217–2180 | 69 |
| 1 to 3 | 657 | 30.3 | 3.87 | 19.0–48.4 | 43 |
| >3 | 505 | 7.2 | 2.40 | 4.0–13.1 | 30 |

The different curves (Figures 5 and 6) are in first approximation parallel. Thus they can be characterised by their median values. The median (termed V50) indicates the volume for which 50% of the tumours have metastasised. The V50 values for the various groups studied and for the overall population are indicated in Table II. For a tumour of a given size, the dissemination probability is less if it belongs to a group with a larger V50 value. The V50 values of three of the groups studied differ significantly ($t$ test, $P<0.001$) from those of the overall population: the group in which the histological grade is equal to 1, and the group with no invaded axillary lymph nodes, have a V50 which is significantly larger than overall; the group with more than three involved lymph nodes has a V50 which is smaller than for the total population. These data are consistent with the results of the analysis of the prognostic factors in this group of patients (Sarrazin *et al.*, 1982). The possible relationship between the histological grading and the number of involved lymph nodes was not taken into consideration in this paper.

### Discussion

This study covers a 19-year period (1954–1972), and it was therefore important to be sure that no notable modification in patient classification procedures had supervened in the meantime (lymph node involvement and histological grade). The technique used to assess the histological grade (Bloom & Richardson, 1957) has not changed since 1958. Concerning the tumours treated before 1958, the histological specimens were reviewed to classify them according to Bloom's grading. The histological technique and the average number of lymph nodes removed and examined have changed little with time (Contesso *et al.*, 1977).

The study is based on clinical measurements and the different classes of tumours are defined at 1 cm diameter. Such a degree of accuracy can appear debatable, however the calculations are made on the mean values of the logarithm of the diameter and the accuracy of the mean is higher than that of the individual values. Moreover, changes in the width of the steps used in classifying the tumour diameters did not affect the result concerning the threshold volume distribution. For example, the slope of the curve and the V50 value of Figure 2 are not modified when the patients are classed with a 2 cm width step in diameter, instead of a 1 cm step.

Twenty-five percent of the patients were entered in the study during the period 1954–1958, during which tumour diameter was not measured directly, but ascertained by reference to a standard object. During this period, the cumulative proportion of patients with metastases is the same as that of the patients of the period 1959–1972 (Figure 2). Moreover, during the two periods, the distributions of tumour volumes are alike, as well as the relationships between the clinical size and the size measured on the surgical specimen (Figure 4). This indicates that the change in the technique of size measurement had no significant impact on its evaluation.

Furthermore, it is well-known that from a statistical point of view, random inaccuracies in the measurement of a variable can mask an existing correlation but cannot create an artefactual relationship.

Most of the previous studies have been carried out using the diameter measured on the surgical specimen because the accuracy of this measurement was supposed to be greater than that of the volume assessed clinically. However, this practice introduces a strong bias in the patient population because the pathological diameter is available for the patients selected for surgical treatment without preoperative radiotherapy. Thus, these patients are not representative of the overall population; in particular most of the patients with metastases at initial presentation are excluded.

However, the information concerning the histological grade and the number of involved lymph nodes is available only for the patients initially treated by surgery, hence the study of the relation between these variables and the V50 was studied on a smaller group of patients whose prognosis was better than average.

Our results are consistent with those reported by Berg & Robbins (1966). They showed that, among other factors, the probability of death by cancer depended, in the long term, on the size of tumours as measured on the surgical specimen. However, these authors did not study this relationship quantitatively and did not attempt to evaluate the dissemination probability. Moreover, this study was carried out among a population from which patients with metastases at diagnosis were excluded. Such a procedure implies that the patients with metastases at diagnosis represent a special group, whereas they only differ from the others by a slightly greater size of the metastases, which allows its detection at the same time as the primary tumour. Nevertheless, we undertook an evaluation of the V50 in the Berg and Robbins series on the basis of their data; the V50 which was found correspond to a tumour diameter of 2.8 cm. When one considers the differences in the patient populations, there is good concordance with our own data.

A combined study of the metastasis probability in relation to tumour size and lymph node invasion was reported by Fisher *et al.* (1969). In this study the follow-up of the patients extended for 5 years only during which an influence of tumour size on the dissemination probability was observed. These results suggested that the influence of mean tumour size was more pronounced for patients bearing more than three involved lymph nodes than for those in the other groups. Our results show that, regardless of the number of invaded nodes, the relationship between size and metastasis probability may be explained by the same model, *viz.* that the initiation of the first metastasis occurs when the tumour volume becomes equal to the threshold volume. Moreover, the parallelism of the curves shows that the type of relationship between size and dissemination probability is the same in the various groups. The difference between the threshold volumes of the groups with different histological grades or number of involved lymph nodes appears to be due to variations in the probability dissemination by unit number of cells.

The earlier detection of distant metastases in patients having several involved lymph nodes deserves additional discussion. The time interval between initial treatment and detection of metastases is influenced by two factors: the growth rate of the metastasis and the time between initiation of the metastasis and initial treatment. These factors have been previously discussed (Tubiana *et al.*, 1975, 1981*a*, Tubiana, 1982). As reported (Tubiana *et al.*, 1975, 1984, Tubiana, 1982) the time interval between relapse and death is not influenced by the number of involved lymph nodes which suggests that the growth rates of metastases are the same regardless of the number of involved lymph nodes (Tubiana, 1982). Moreover, the labelling indices of primary breast tumours do not differ in groups of patients with various numbers of involved nodes (Tubiana *et al.*, 1981*b*). Therefore, it was previously inferred that the earlier detection of metastases in patients with several involved lymph nodes was probably due to an earlier initiation of metastasis, in other words, to a smaller threshold volume. This idea is supported by present data which show that the dissemination probability curves (Figure 5) are almost parallel and that the median volume (V50) for which 50% of the tumours have disseminated is smaller in patients with several involved lymph nodes (Table II).

The differences in the duration of median delay between diagnosis and detection of metastases (Table II) provide information of interest. Our data confirm the prognostic significance of histological grading (Contesso *et al.*, 1977, Sarrazin *et al.*, 1982), and stress its influence on the dissemination probability. They indicate that in the long term, the distinction between the grades 2 and 3 is not important, from the point of view of metastasis probability. However, the delays between diagnosis and detection of metastases differ, suggesting a more rapid growth rate in grade 3 than in grade 2 tumours. This results is in agreement with our previous measurement of labelling indices (Tubiana *et al.*, 1981*b*, 1984).

The estimation of the primary tumour volume at the initiation of metastasis in osteosarcomas, fibrosarcomas and seminomas was attempted by Breur (1976) and in breast cancer by Igot & Le Gal (1968). The method used consisted of extrapolating the metastases growth curve backwards in order to determine at what moment the metastasis contained one single cell. Though these studies were carried out on different types of tumours, their results suggested that metastases are initiated very early in the life of these tumours, and that the percentage of metastasis initiated after the tumours reach a detectable size is very low. These conclusions conflict with our present data which show that the primary tumours are relatively large when metastases are initiated. Moreover they implied that earlier tumour treatment should result in only a negligible reduction in the percentage of metastases (Igot & Le Gal 1968), whereas it has been shown that, in patients with breast cancers, an early detection of tumours results in a considerable decrease of cancer mortality, due to a smaller metastasis incidence (Strax 1978, Thomas *et al.*, 1977, Shapiro *et al.*, 1982).

The discrepancy between our data and the above-mentioned calculations can be attributed to the hypotheses supporting the previous models. The main hypotheses underlying those models were: (i) tumour growth curves are of the exponential type and (ii) the growth rate of a tumour and its metastases are equal. Present knowledge concerning the pattern of human tumour growth do not warrant these hypotheses. In particular, the doubling times of primary tumours are on the whole longer than those of metastases (Charbit *et al.*, 1971; Steel 1977). Thus, the discrepancy between our data and the results obtained by the backwards extrapolation of the metastases growth curve might be explained by the inadequacy of the model used. This will be further discussed in a subsequent article.

### References

BERG, J.W. & ROBBINS, G.F. (1966). Factors influencing short and long term survival of breast cancer patients. *Surg. Gynecol. Obst.*, **122**, 1311.

BREUR, K. (1976). Conference on the "Biological behaviour of tumour and chronobiology in tumours". Oslo (unpublished, quoted in Tubiana 1982).

BLOOM, H.J.G. & RICHARDSON, W.W. (1957). Histological grading and prognosis in breast cancer. A study of 1409 cases of which 359 have been followed 15 years. *Br. J. Cancer*, **11**, 359.

CHARBIT, A., MALAISE, E.P. & TUBIANA, M. (1971). Relation between the pathological nature and the growth rate of human tumors. *Eur. J. Cancer*, **7**, 307.

CONTESSO, G., ROUESSE, J., PETIT, J.Y. & MOURIESSE, H. (1977). Les facteurs anatomo pathologiques du pronostic des cancers du sein. *Bull. Cancer* (*Paris*), **64**, 525.

COLLINS, V.P., LOEFFLEY, K.R. & TIVEY, H. (1956). Observations on growth rates of human tumors. *Am. J. Roentgen.*, **76**, 988.

FINNEY, D.J. (1964). *Statistical Method in Biological Assay* (2nd edition). London, Charles Griffin & Co.

FISHER, B., SLACK, N.H., BROSS, I.D.J., & cooperative investigators. (1969). Cancer of the breast: size of neoplasm and prognosis. *Cancer*, **24**, 1071.

IGOT, J.P. & LE GAL, Y. (1968). Age des adenopathies métastatiques dans le cancer mammaire. *Ann. Anat. Pathol.*, **13**, 449.

KAPLAN, E.L. & MEIER, P. (1958). Non parametric estimations from incomplete observations. *J. Am. Statist. Assoc.*, **53**, 457.

LACOUR, J., JURET, P. & SARRAZIN, D. (1968). Protocole schématique de traitement des cancers du sein à l'Institut Gustave Roussy. *Rev. Prat.* (Paris), **18**, 3595.

OKELL, C.C. (1964). On the quantitative study of tumours. *J. Pathol. Bacteriol.*, **88**, 303.

SARRAZIN, D., LÊ M., MOURIESSE H. & 4 others. (1982). Radiotherapeutic studies on breast cancer at Villejuif. *Cancer Bull.*, **34**, 242.

SHAPIRO, S., VENET, W., STRAX, P., VENET, L. & ROESER, R. (1982). Ten- to fourteen-year effect of screening on brast cancer mortality. *J. Natl Cancer Inst.*, **69**, 349.

STEEL, G.G. (1977). *Growth Kinetics of Tumours*. Oxford University Press.

STRAX, P. (1978). Evaluation of screening programs for the early diagnosis of breast cancer. *Surg. Clin. North Am.*, **58**, 667.

THOMAS, L.B., ACKERMAN, L.V., McDIVITT, R.W., HANSON, T.A.S., HANKEY, B.F. & PROROK P.C. (1977). Report of NCI Ad Hoc Pathology Working Group to Review the Gross and Microscopic Findings of Breast Cancer Cases in the HIP Study. *J. Natl Cancer Inst.*, **59**, 497.

TUBIANA, M., CHAUVEL, P., RENAUD, A. & MALAISE, E.P. (1975). Vitesse de croissance et histoire naturelle du cancer du sein. *Bull. Cancer* (Paris), **64**, 341.

TUBIANA, M., VALLERON, A.J. & MALAISE, E. (1981*a*). The natural history of human breast cancer. In: *New frontiers in Mammary Pathology*. (Eds. Hollman *et al.*) New York, p. 239.

TUBIANA, M., PEJOVIC, M.J., RENAUD, A. & 4 others. (1981*b*). Kinetic parameters and the course of the disease in breast cancer. *Cancer*, **47**, 937.

TUBIANA, M. (1982). Cell kinetics and radiation oncology. *Int. J. Radiat. Oncol. Biol. Phys.*, **8**, 1471.

TUBIANA, M., PEJOVIC, M.H., CHAVAUDRA, N., CONTESSO, G. & MALAISE, E.P. (1984). Long term prognostic significance of the labelling index in breast cancer. *Int. J. Cancer* (In press).